# Effect of Volatile Organic Chemicals in *Chrysanthemum indicum* Linné on Blood Pressure and Electroencephalogram

**DOI:** 10.3390/molecules23082063

**Published:** 2018-08-17

**Authors:** Da-Som Kim, Young-Min Goo, Jinju Cho, Jookyeong Lee, Dong Yeol Lee, Seung Mi Sin, Young Sook Kil, Won Min Jeong, Keon Hee Ko, Ki Jeung Yang, Yun Geun Kim, Sang Gon Kim, Kiseong Kim, Young Jun Kim, Jae Kyeom Kim, Eui-Cheol Shin

**Affiliations:** 1 Department of Food Science, Gyeongnam National University of Science and Technology, Jinju, Gyeongnam 52725, Korea; kim94dasom@naver.com (D.-S.K.); aacho7@daum.net (J.C.); tracylee0911@gmail.com (J.L.); 2 Gyeongnam Oriental Medicinal Herb Institute, Sancheong 52215, Korea; dudals1109@naver.com (Y.-M.G.); 7560112@naver.com (D.Y.L.); sksskdi714@hanmail.net (S.M.S.); miohkil@naver.com (Y.S.K.); wonmin5618@gmail.com (W.M.J.); kosajang83@naver.com (K.H.K.); yang2229@gnherb.or.kr (K.J.Y.); koreanol@hanmail.net (Y.G.K.); sen600@hanmail.net (S.G.K.); 3 Department of Bio and Brain Engineering, Korea Advanced Institute of Science and Technology (KAIST), Daejeon 34141, Korea; iames@kaist.ac.kr; 4 Department of Food and Biotechnology, Korea University, Sejong 30019, Korea; yk46@korea.ac.kr; 5 School of Human Environmental Sciences, University of Arkansas, Fayetteville, AR 72701, USA; jkk003@uark.edu

**Keywords:** *Chrysanthemum indicum* Linné, essential oil, volatiles, electroencephalogram, blood pressure

## Abstract

This study identified the volatile organic compounds in the essential oils that are extracted from *Chrysanthemum indicum* Linné (*C. indicum* Linné) and investigated the effects of the inhalation of these compounds. We detected a total of 41 volatile organic compounds, including 32 hydrocarbons, four acids, three alcohols, two ketones, and one aldehyde. In a sniffing test, seven types of volatile organic compounds were identified. Furthermore, the volatile organic compounds in *C. indicum* Linné that were identified were found to be derived from 1,8-cineole and camphor. After inhalation of the essential oils, the subjects’ systolic blood pressure and heart rate decreased. This indicates that inhalation of the essential oils extracted from *C. indicum* Linné provides mental and physical relaxation. We examined the changes in electroencephalogram findings that are observed after *C. indicum* Linné essential oil inhalation. An increase in theta and alpha waves, which usually appear during relaxation, as well as a decrease in beta and gamma waves, which appear during brain activity such as excessive attention, were noted. These results indicate that *C. indicum* Linné essential oil inhalation helps to reduce blood pressure and may provide mental and physical relaxation.

## 1. Introduction

In modern life, we are inundated with a diverse range of information as the complex society we live in rapidly changes, exposing us to various mental, physical, and environmental stresses. The pace at which advances in civilization proceed is not determined by what most people consider the pace of happiness, leading to a reality in which people feel less happy and less at peace [1]. This stress causes depressive emotions, which manifest in several forms, irrespective of age or gender. Researchers agree that these depressive emotions are ultimately caused by stress. If stress is persistent, it can lead to impairments in brain and neural development, anhedonia, irritable colon syndrome, impaired attention, loss of appetite, and sleep disorders [2,3]. The medications that are currently used to treat depression include selective serotonin reuptake inhibitors (SSRIs) and serotonin and noradrenergic reuptake inhibitors (SNRIs). However, as the pathways underlying depression have not been clearly elucidated, pharmacological treatment always carries a risk of adverse effects [4].

The essential oils that are obtained from plants are essential secondary metabolites for survival and come in the form of low-boiling point oils that are stored in specific extracellular areas, differentiated from the plant epidermis or leaf body. These substances are low-molecular weight liquid mixtures that readily evaporate in air and can be recognized through human olfaction [5]. Plant-derived essential oils are not single chemical substances. They are highly volatile terpene compounds that can be classified, depending on their functional group, into alcohols, aldehydes, ketones, ethers, esters, acids, and oxides [6]. Compared with other substances, essential oils are only present in small quantities; however, they are considered important while determining product preferences and quality, and plant strains are selected based on their essential oil content [7,8,9]. The methods that are used to purify essential oils from natural materials include simultaneous steam distillation extraction (SDE), solvent extraction, and solid-phase microextraction (SPME); SDE is commonly used for relatively heat-stable samples, while SPME has been increasingly used recently, because it is possible to easily pretreat a small amount of the sample without using an organic solvent [10,11]. 

*Chrysanthemum indicum* (*C. indicum*) Linné is a perennial plant of the Compositae family; in South Korea, wild *C. indicum* Linné are found in several mountainous regions in the south. These plants flower from June to October, with yellow petals and flower buds that are 1.5 cm in length and gathered at the end of the stems. The flower, which is the part of the plant that is typically used in traditional Korean medicine, has an excellent aroma, called the aroma compositae. In addition to its medicinal usage, due to its pleasant scent, these flowers can also be added to rice cakes, used as a fragrance, or used in making chrysanthemum wine [12]. In traditional Korean medicine, *C. indicum* Linné is used for antipyretic and anti-inflammatory effects, blood pressure lowering, and headache relief [13,14]. Recently, several studies have demonstrated that *C. indicum* Linné has antibacterial, anti-inflammatory, immunomodulatory, antioxidative, and anticancer properties [15,16,17,18,19].

Electroencephalography (EEG) is the recording of amplified intracellular electric signals in the cerebral cortex taken from an intact scalp. EEG results are plotted with the electric potential on the vertical axis and time on the horizontal axis. Compared with other brain imaging techniques, EEG is able to provide rapid and accurate information in real-time. EEG measures activity in the central nervous system, recording the spontaneous electrical activity of the brain using a number of electrodes that are attached to the scalp. EEG reflects the sum of electrical activity occurring simultaneously in tens of thousands of neurons; EEG usually refers to spontaneous brain waves and is thus differentiated from evoked potentials, which occur in the nervous system following a stimulus [20,21]. EEG is a simple, economic, convenient-to-use method for functional brain imaging. This technique is, however, limited by its low spatial resolution [22]. Recently, quantitative EEG (QEEG) has been developed, using a computer to perform spectrum analysis of the recorded brainwave signals, in order to provide information that cannot be obtained from analysis with the naked eye [23].

The downstream signaling of the inhalation of essential oils is as follows: aromatic particles are inhaled through the nose where they stimulate olfactory receptors. This stimulus is conveyed to the limbic system, where it evokes an emotional, instinctive response; in turn, this stimulates the autonomic nervous system, which modulates the heart rate, blood pressure, breathing, reproduction, memory, and stress response. Meanwhile, aromatic particles that are absorbed by the skin enter the bloodstream, are absorbed by the pulmonary alveoli, and spread across the whole body, reacting chemically through hormones and enzymes [24]. Based on these mechanisms of the inhalation of essential oils, we explored the changes in EEG recordings made after the inhalation of essential oils from *C. indicum* Linné.

## 2. Materials and Methods

### 2.1. Materials

The *C. indicum* Linné flowers used in this experiment were collected from Gyeongnam, Korea, in 2017. In order to preserve the volatile organic compounds (VOCs), the specimens were stored at −20 °C until they were used.

### 2.2. Sniffing Test and Analysis of VOCs in C. indicum Linne Essential Oil

The sample (*C. indicum* Linné flowers) of 500 g placed in 5 L hexane was extracted at room temperature for 2 days and filtered. The filtered extract was mixed with 1L ethanol and dissolved completely. The solution was then left in a −10 °C refrigerator for 5 h for second extraction. The extract was centrifuged at 7000 rpm for 10–20 min, and the supernatant was concentrated using a rotary evaporator (BUCHI Co., New Castle, DE, USA). The extraction yield in hexane was 2.2%. We performed headspace analysis of the *C. indicum* Linné extract using a SPME fiber coated in 100 μm polydimethylsiloxane (PDMS; Supelco Co., Bellefonte, PA, USA). After placing 1 g of the sample into a gas collecting tube and sealing it with an aluminum cap, the SPME fiber was injected into the tube, heated to 50 °C, and exposed to the sample vapor for 30 min to adsorb the VOCs. Thereafter, for analysis of the individual VOCs that were adsorbed by the SPME fiber, the fiber was subjected to gas chromatography using a mass detector.

The VOCs were analyzed by gas chromatography-mass spectrometry (GC/MSD; Agilent 7890A and 5975C, Agilent Technologies, Santa Clara, CA, USA). We used an HP-5MS column (30 m × 0.25 mm i.d. × 0.25 μm film thickness). The oven temperature was set to 40 °C for 5 min before increasing by 5 °C/min up to a temperature of 200 °C. The injector temperature was set to 220 °C to separate the VOCs from the SPME fiber. The flow rate of helium, the carrier gas, was 1.0 mL/min, and the splitless injection mode was selected. The individual compounds that were isolated in the total ionization chromatogram (TIC) of the sample were identified using the mass spectra that were provided in version 12 of the NIST (National Institute of Standards and Technology) Library. The relative concentration of each VOC was calculated by comparing the peak area of each compound with the concentration and peak area of pentadecane (C15:0), which acted as the reference standard [25].

In the sniffing test, subjects used an olfactory detection port with a heated mixing chamber (ODP 3, Gerstel, Inc., Linthicum, MD, USA) attached to the GC/MSD to smell individual VOCs that were isolated by GC/MS. To account for individual differences in the intensity of smell and the decreased olfactory sensitivity over time, three experienced experimenters participated in the same experiment and inspected the intensity of each VOC [26].

### 2.3. Measurement of Blood Pressure and EEG

We recorded the changes in blood pressure, heart rate, and EEG in 10 adult male and female subjects after the inhalation of the *C. indicum* Linné essential oil. Before starting the EEG experiment, we confirmed that the subjects had no history of psychiatric disease, continual medication, or nose surgery. Before the experiment, the subjects were instructed to abstain from irritating foods, smoking, and alcohol. A blood pressure monitor (HEM-7322, Omron Healthcare Korea Co., Seoul, Korea) was used to measure the blood pressure and heart rate from the left arm of the subject while they were at rest. The measurements were taken twice before and twice after the essential oil inhalation, and the data from each individual was analyzed for significant changes [27].

In order to measure the changes in brainwaves following essential oil inhalation, a polygraph system (Bios-S-24, Biobrain Inc., Daejeon, Korea) was used; disc-shaped electrodes were attached to the scalp of each subject using electrode paste (ElfixZ-401CE, Nihon Koden Co., Tokyo, Japan). Following the international standard 10-20 electrode system, electrodes were placed in 10 locations corresponding to the prefrontal lobe (Fp1, Fp2), the frontal lobe (F3, F4), the temporal lobe (T3, T4), the parietal lobe (P3, P4), and the occipital lobe (O1, O2); reference electrodes were placed behind the ears, and the ground electrode was placed at the back of the neck. Brainwaves were measured for 3 min while the subject was at rest with closed eyes (control). Measurements were then made for another 3 min while the subject inhaled the essential oil. Using a brainwave measurement program (BioScan, Biobrain Inc., Daejeon, Korea), we collected data in real-time throughout the experiment. The collected data was then processed using a brainwave analysis program (BioScan, Biobrain Inc., Daejeon, Korea) and a batch processing program (BioScan-Batch, Biobrain Inc., Daejeon, Korea). A brain mapping program (Brain Map3D, Laxtha Co., Daejeon, Korea) was used to map the mean EEG measurements to the subject’s brain during the experiment [28,29]. This study was approved by an institutional review board (P01-201804-11-001) and consent was obtained from each subject before beginning the experiment.

### 2.4. Statistical Processing

For all of the results presented in this study, we used SAS version 9.2 (SAS Institute Inc., Cary, NC, USA) to calculate the significance using paired T-tests (*p* < 0.05).

## 3. Results

### 3.1. VOC Analysis and Sniffing Test for C. indicum Linne

Table 1 shows the VOCs that were contained in the sample of *C. indicum* Linné that was used in this study. We identified a total of 42 VOCs, with 32 hydrocarbons, four acids, three alcohols, two ketones, and one aldehyde. Individual VOCs included camphor (621.08 ± 18.51 μg/mL), germacrene D (117.43 ± 16.62 μg/mL), bornyl acetate (86.99 ± 17.21 μg/mL), 1,8-cineole (66.18 ± 8.59 μg/mL), and β-ocimene (60.77 ± 8.63 μg/mL). 

In addition to measuring the concentrations of these VOCs, we also used a sniffing test to classify the VOCs that were associated with *C. indicum* Linné by intensity. Of the 7 VOCs that could by identified by smell, 1,8-cineole gave a very strong sense of *C. indicum* Linné, while camphor showed a low strength similarity to our sample of *C. indicum* Linné. In general, the odor image of the *C. indicum* Linné that was used in this study was considered to be mostly due to 1,8-cineole and camphor. In a study by Shin et al., 45 VOCs were reported to be present in *C. indicum* Linné, while camphor and 1,8-cineol were reported to be the main VOCs [12]. These results are consistent with the results of the current study. In the literature, 1,8-cineole is reported to be a VOC that is commonly found in the essential oils of *Eucalyptus globulus* and *Laurus nobilis*. This compound is used in ointments to treat coughs, muscular pain, and rheumatism [30]. In a study by Miyazawa, 1,8-cineole was detected in the urine of people who had taken cold medicine, and 1,8-cineole was reported to be used as a major marker in urine [31]. Meanwhile, camphor is known to be used commercially in moth repellent and antiseptic agents. Similar to 1,8-cineole, there are also reports of camphor being used to treat rheumatic pain and coughs [30].

### 3.2. Effect of Inhalation of the VOCs in C. indicum Linné on Blood Pressure and EEG

We recorded the changes in heart rate and blood pressure in 10 adult male and female subjects after inhalation of VOCs using *C. indicum* Linné essential oil (Table 2). The heart rate decreased after VOC inhalation in eight out of 10 subjects, and this decrease was significant in five subjects (*p* < 0.05). The systolic blood pressure decreased after VOC inhalation in nine out of 10 subjects, and this decrease was significant in seven subjects (*p* < 0.05). In contrast, the diastolic blood pressure showed no consistent response pattern. However, there was a significant decrease in the diastolic blood pressure of four subjects (*p* < 0.05). These results suggest that inhalation of the VOCs in *C. indicum* Linné lowers systolic blood pressure and has a relaxing effect on heart rate.

Nasally inhaled VOCs are conveyed via olfactory receptors to the limbic system, where they cause an emotional, instinctive response; in turn, they stimulate the autonomic nervous system, modulating the heart rate, blood pressure, breathing, reproduction, memory, and stress response. Additionally, the VOCs that enter through the skin are transported in the blood stream and absorbed by the pulmonary alveoli, spreading to the whole body to take part in various chemical reactions [24]. In a study by Jung et al., the majority of the 29 participants they tested showed a decrease in heart rate after inhaling the VOCs from ylang-ylang [27]. Kikuchi et al. observed a decrease in the heart rate of participants following inhalation of the VOCs from rose oil and concluded that rose oil inhalation had a relaxing effect [32]. Kovar et al. observed an increase in blood 1,8-cineole levels after rosemary oil inhalation in a mouse model and found that this increase correlated with locomotor stimulation [33]. Sayorwan detected a decrease in the heart rate of humans following sweet almond oil inhalation, while rosemary oil inhalation was, conversely, associated with a temporary increase in heart rate [34]. Based on these studies, we can deduce that inhalation of plant-derived essential oils does not induce a decrease in heart rate but that the actions of these oils are mediated by the compounds that they contain. It will be necessary to investigate the VOCs that cause a decrease in heart rate in further studies.

**Figure 1:**
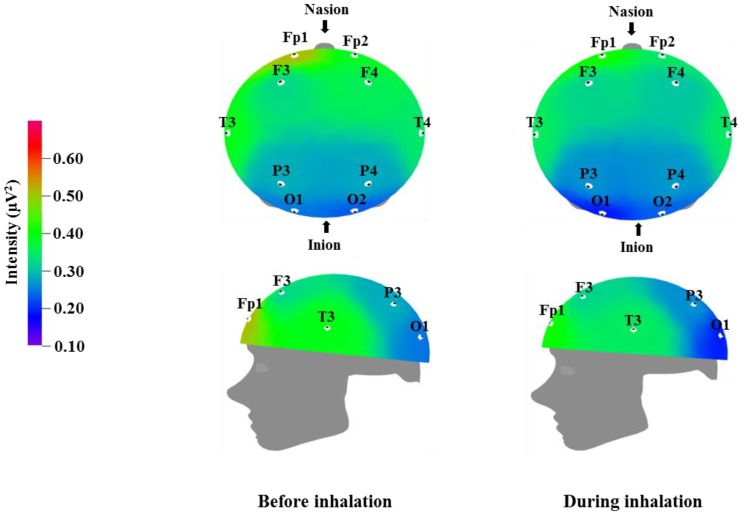
Relative theta wave intensity between before and during inhalation of VOCs from *C. indicum* Linné.

**Figure 2:**
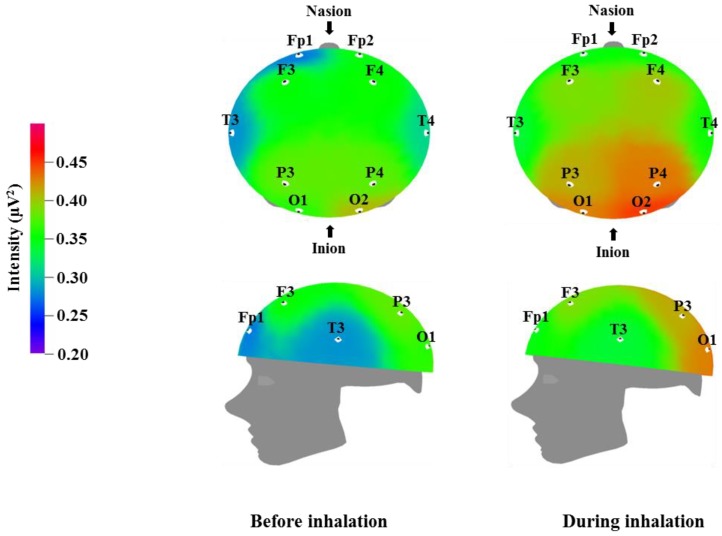
Relative alpha wave intensity between before and during inhalation of VOCs from *C. indicum* Linné.

**Figure 3:**
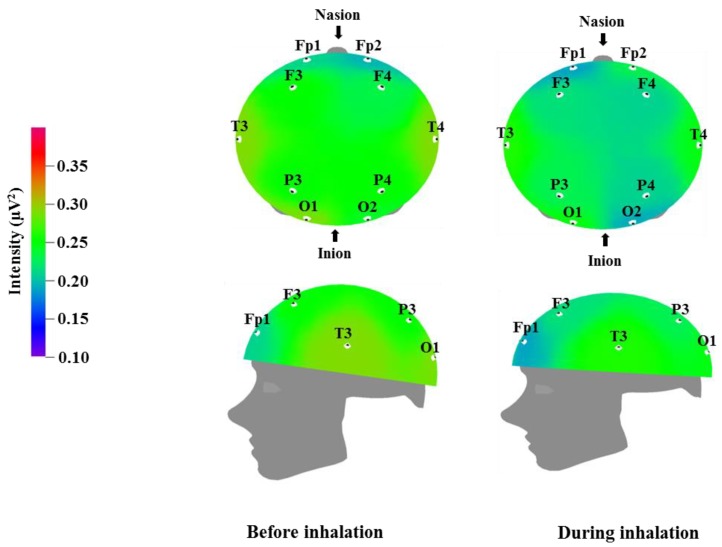
Relative beta wave intensity between before and during inhalation of VOCs from *C. indicum* Linné.

**Figure 4:**
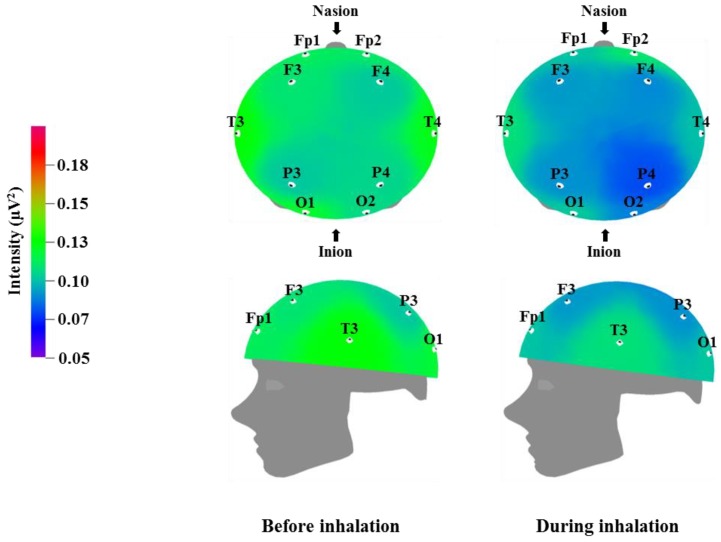
Relative gamma wave intensity between before and during inhalation of VOCs from *C. indicum* Linné.

We used EEG to measure the brainwaves of participants before and after the inhalation of the VOCs from *C. indicum* Linné; the results are displayed in Table 3, Table 4, Table 5 and Table 6, while the brain mapping is shown in Figure 1, Figure 2, Figure 3 and Figure 4. Different regions of the cortex fulfill different roles. EEG allows us to examine different regions of the brain where electrodes are attached. Fp1 and Fp2 were placed over the prefrontal cortex, which is involved in attention and logical reasoning; abnormalities in attention are highlighted by the responses in the area of Fp1, while reasoning and impulse control are associated with the responses in the area of Fp2. F3 and F4 were located over the frontal cortex, with each of these regions affecting the contralateral side of the body. These regions are involved in thought processes, including goal selection, decision making, social judgments, and initiation of behavior; in cases of hyperactivity or impairment in the frontal area function, defects are observed in attention, awareness, motor function, affect, and memory [35]. T3 and T4 were located over the temporal cortex, which is responsible for receiving and processing auditory and olfactory information, and is also involved in linguistic understanding, stimulation, and control of emotion. Injury to the temporal area is known to manifest in auditory defects, aphasia, and impaired stimulus recognition [36]. P3 and P4 were located over the parietal cortex, which is associated with sensory and motor function. The parietal cortex is involved in receiving and verifying sensory information and affects physical awareness, left–right discrimination, abstract thought, and interpretation of mathematical symbols; abnormalities in the parietal area are known to cause agnosia and impaired awareness of physical sensation [37]. O1 and O2 were placed over the occipital cortex, which is responsible for visual function. Injury in this area is known to lead to impaired visual cognition. In addition, when the temporal lobe and occipital lobe are both injured, this can result in visual field defects or cortical blindness accompanied by object agnosia, achromatopsia, and visual agnosia [38].

Table 3 and Figure 1 show the recorded changes in the relative theta waves; an increase was observed at eight electrode sites: Fp2, F3, F4, T3, T4, P3, P4, and O2. Theta waves are in the 4–8 Hz frequency band. These waves are usually observed in adults during the early sleep stages, when the individual begins to feel drowsy, and are known to also increase when an individual is thinking of new ideas. Theta waves are associated with the hippocampus, which plays an important role in memory in humans. Better memory has been observed for events occurring at times of increased theta wave activity [39]. Given that theta waves are detected during peaceful meditation, the overall increase in theta waves that was observed after essential oil inhalation may be related to mental and physical relaxation. 

Table 4 and Figure 2 show the recorded changes in the relative alpha waves; an increase was observed at nine electrode sites: Fp1, F3, F4, T3, T4, P3, P4, O1, and O2. Alpha waves are in the 8–12 Hz frequency band, and are commonly observed in a state of rest, such as meditation or peaceful relaxation. An increase in alpha wave activity indicates a decrease in activity in the corresponding cortical region. Alpha wave increases occur when the subject feels physical and mental relaxation and comfort and can be considered to be correlated with the reduced blood pressure and heart rate shown in Table 2. Given that alpha wave activity typically becomes weaker during excited states or cognitive activity, the overall alpha wave response observed following essential oil inhalation is suggestive of mental relaxation. Similarly, Sugano reported increases in alpha wave activity following the inhalation of α-pinene, 1,8-cineole, lavender, sandalwood, musk, and eucalyptus [40]. Lee et al. detected various brainwave changes following the inhalation of citrus, lavender, and floral flavors, with lavender in particular showing an increase in alpha wave activity in the occipital region [41]. Lorig reported that pleasant odors promote increased alpha waves, whereas unpleasant odors cause a decrease in alpha waves [42]. Pleasant odors were also found to result in different breathing patterns; specifically, pleasant odor inhalation was associated with deeper breathing. This change in breathing pattern could be one factor that contributes to the mental and physical relaxation that appears to be induced by essential oil inhalation. Our results are consistent with the results of previous studies, which have reported that 1,8-cineole and methyl jasmonate are factors that could cause increases in theta and alpha waves similar to those described in Table 3 and Table 4 [43]. In particular, the effects of 1,8-cineole have been demonstrated in both human and animal studies [33,44].

Table 5 and Figure 3 show the recorded changes in the relative beta waves; a decreasing trend was observed at nine electrode sites, excluding Fp2: Fp1, F3, F4, T3, T4, P3, P4, O1, and O2. Beta waves are in the 12–30 Hz frequency band and can be categorized further into low beta waves and high beta waves. Low beta wave activity is known to increase awareness. Excessive high beta wave activity can be a major cause of anxiety in subjects, and highly anxious subjects who are overly cautious can show strong high beta wave activity [39]. Recent research has demonstrated that high beta wave activity is associated with migraines [45]. The results in Table 3 and Table 4 show a trend towards a negative correlation between increased theta and alpha wave activity, which is associated with relaxation and attention, and decreased beta wave activity, which is associated with anxiety and nervousness.

Table 6 and Figure 4 show the recorded changes in the relative gamma waves; similar to the relative beta wave results, a decrease was observed in the gamma waves at nine electrode sites, excluding Fp2: Fp1, F3, F4, T3, T4, P3, P4, O1, and O2. Gamma waves are in the 31–50 Hz frequency band and represent the highest frequency band for brainwaves. Gamma waves usually appear during focused attention during complex problem solving or while using multiple cortical areas to think of an answer; thus, they are closely related to learning [39]. However, excessive gamma wave activity can cause cortical fatigue. Similar to the relative beta wave activity shown in Table 5 and Figure 3, the decrease in gamma wave activity observed after inhalation of *C. indicum* Linné essential oil is thought to be related to brain relaxation and the decrease in heart rate and blood pressure.

## 4. Discussion

In this study, we analyzed the VOCs in *C. indicum* Linné and identified 42 different VOCs through GC/MS. In an olfactory analysis, the compounds that were determined to best represent the scent of *C. indicum* Linné were 1,8-cineole and camphor. We measured the heart rate and blood pressure changes following inhalation of the VOCs from *C. indicum* Linné, including 1,8-cineole and camphor, and observed decreases in both the heart rate and blood pressure. We also conducted EEG to examine the changes in theta, alpha, beta, and gamma waves after VOC inhalation; we observed an increase in theta and alpha wave activity on inhalation of the VOCs in *C. indicum* Linné. Such changes are thought to be associated with mental and physical relaxation. Conversely, the high-frequency beta and gamma wave activities, which are associated with brain activity, awareness, focused attention, and anxiety, were decreased. These results demonstrate that, even without direct ingestion of plant materials, inhalation of the essentials oils that are extracted from *C. indicum* Linné alone can induce changes in heart rate, blood pressure, and EEG activity. In the future, we hope that our study can be used as fundamental research to support the use of essential oils from various plants as relaxation-inducing agents

## Figures and Tables

**Table 1:** Volatile organic chemicals (VOCs) in *Chrysanthemum indicum* Linné.

Compounds	Retention Time (min)	Retention Index	Concentration (μg/mL)	Odor Intensity ^1^	Odor Description
**Acids**					
2-Methyl butanoic acid	10.77	892	0.81 ± 0.08		
Tiglic acid	12.38	942	0.38 ± 0.15		
Decanoic acid	25.87	1400	4.53 ± 0.29		
Ethyl ester decanoic acid	26.35	1420	2.27 ± 0.27		
**Alcohols**					
1,8-Cineole	16.35	1063	66.18 ± 8.59	4	Chrysanthemum indicum
1,7,7-Trimethyl-2,2,1-heptan-2-ol	20.60	1203	45.19 ± 2.05	3	Sharp
2-Methyl-5-1-methyl ethyl phenol	24.11	1333	1.42 ± 0.37		
**Aldehyde**					
2-Methyl-3-phenyl propanal	22.47	1272	0.73 ± 0.11		
**Hydrocarbons**					
1,3-Dimethyl benzene	11.75	921	0.11 ± 0.02		
Camphene	13.58	977	3.88 ± 0.64		
Trimethyl benzene	14.05	990	1.88 ± 1.62		
1-Ethyl methyl benzene	14.09	991	0.78 ± 0.38		
Sabinene	14.41	999	1.53 ± 1.21	1	Paint
Phellandrene	15.42	1033	4.35 ± 2.95		
α-Terpinene	15.81	1046	4.53 ± 1.32		
1-Methyl-3-1-methyl ethyl benzene	16.06	1054	6.73 ± 5.08		
β-Ocimene	16.75	1075	60.77 ± 8.63		
1,2-Diethyl benzene	17.01	1083	1.22 ± 0.03	2	Solvent
Sabinene hydrate	17.45	1096	3.72 ± 0.14		
4-Ethyl-1,2-dimethyl benzene	17.72	1105	1.41 ± 0.03		
Camphor	20.10	1187	621.08 ± 18.51	1	Chrysanthemum indicum
Azulene	21.01	1219	4.81 ± 0.05	4	Hospital
Dodecane	21.18	1225	2.00 ± 0.01		
3,4-Dimethoxy toluene	22.34	1268	2.78 ± 0.52		
1,2,4-Triethyl benzene	22.40	1269	1.32 ± 0.28		
Dimethyl indan	22.70	1280	1.92 ± 1.63	2	Pencil
Bornyl acetate	23.77	1320	86.99 ± 17.21		
Tridecane	23.86	1324	2.47 ± 0.31		
Neryl acetate	25.57	1389	2.95 ± 0.42		
α-Copaene	26.05	1408	4.20 ± 0.94		
Biphenyl	26.11	1410	0.70 ± 0.09		
Tetradecane	26.44	1424	20.07 ± 4.16		
Caryophyllene	27.24	1457	36.18 ± 8.46		
Germacrene D	27.40	1463	117.43 ± 16.62		
β-Farnesene	28.01	1488	38.33 ± 9.49		
Aroma dendrene	28.40	1503	3.32 ± 3.66		
β-Selinene	28.56	1510	26.05 ± 5.36		
α-Farnesene	29.11	1534	4.73 ± 2.06		
β-Bisabolene	29.20	1538	3.76 ± 0.74		
β-Sesquiphellandrene	29.61	1555	24.89 ± 4.34		
**Ketones**					
3-Cyclohepten-1-one	9.73	864	0.05 ± 0.02		
d-Carvone	22.57	1276	2.21 ± 0.64		

Concentration results were expressed mean ± standard deviation. ^1^ Odor intensity was measured by panelists using a gas chromatography (GC)-olfactory system (intensity 1: low; 2: medium; 3: strong; and 4: very strong).

**Table 2:** Heart rate, systolic pressure, and diastolic pressure on the pre- and post-inhalation of volatile organic chemicals (VOCs) in *Chrysanthemum indicum* Linné.

Subject	Heart Rate (beats/min)		Systolic Pressure (mm Hg)		Diastolic Pressure (mm Hg)	
	Pre	Post	Pre	Post	Pre	Post
1	97.5 ± 0.7	90.5 ± 3.5 *	116.5 ± 5.4	109.0 ± 1.4 *	79.5 ± 2.1	73.0 ± 0.1 *
2	83.0 ± 6.5	71.0 ± 0.1 *	105.5 ± 3.5	95.0 ± 0.1 *	61.0 ± 0.1	58.5 ± 3.5
3	81.5 ± 2.5	75.5 ± 2.1 *	121.0 ± 0.1	118.5 ± 2.1	86.0 ± 0.1	77.5 ± 0.7 *
4	72.5 ± 0.7	72.5 ± 2.1	97.5 ± 0.7	99.5 ± 7.8	69.5 ± 3.5	69.5 ± 7.8
5	92.5 ± 2.1	86.0 ± 1.4 *	121.5 ± 0.7	120.5 ± 0.7	71.0 ± 1.4	72.0 ± 0.1
6	87.5 ± 2.1	77.5 ± 2.1 *	116.0 ± 1.4	111.5 ± 0.7 *	58.5 ± 2.1	63.5 ± 3.5
7	76.5 ± 0.7	74.0 ± 1.4	128.5 ± 4.9	119.5 ± 2.1 *	79.0 ± 1.4	74.0 ± 1.4 *
8	76.5 ± 2.1	78.5 ± 3.5	106.5 ± 3.5	99.6 ± 0.7 *	67.5 ± 7.8	66.0 ± 0.1
9	80.5 ± 2.1	76.0 ± 4.2	127.5 ± 2.1	120.5 ± 0.7 *	70.0 ± 4.2	67.5 ± 2.1
10	72.5 ± 3.5	76.0 ± 0.1	134.0 ± 5.7	126.5 ± 2.1 *	63.0 ± 4.2	55.5 ± 4.9 *

Data present mean ± standard deviation. * Corresponds the significant difference between pre and post at *p* < 0.05 by paired T-test.

**Table 3:** Effect of inhalation of volatile organic chemicals (VOCs) from *Chrysanthemum indicum* Linné on relative theta wave.

Site	Relative Theta Activity (μV^2^)			
	Inhalation	Mean	Standard Error	*p*-Value
Fp1: Left prefrontal	Before	0.411	0.072	0.694
	During	0.387	0.073	
Fp2: Right prefrontal	Before	0.333	0.071	0.933
	During	0.339	0.050	
F3: Left frontal	Before	0.295	0.045	0.561
	During	0.324	0.049	
F4: Right frontal	Before	0.312	0.047	0.916
	During	0.317	0.052	
T3: Left temporal	Before	0.334	0.067	0.912
	During	0.339	0.064	
T4: Right temporal	Before	0.305	0.051	0.466
	During	0.337	0.056	
P3: Left parietal	Before	0.272	0.048	0.689
	During	0.289	0.053	
P4: Right parietal	Before	0.273	0.046	0.633
	During	0.298	0.052	
O1: Left occipital	Before	0.252	0.044	0.938
	During	0.248	0.053	
O2: Right occipital	Before	0.244	0.056	0.650
	During	0.272	0.060	

**Table 4:** Effect of inhalation of volatile organic chemicals (VOCs) from *Chrysanthemum indicum* Linné on relative alpha wave.

Site	Relative Alpha Activity (μV^2^)			
	Inhalation	Mean	Standard Error	*p*-Value
Fp1: Left prefrontal	Before	0.283	0.047	0.229
	During	0.323	0.051	
Fp2: Right prefrontal	Before	0.370	0.072	0.697
	During	0.342	0.046	
F3: Left frontal	Before	0.374	0.050	0.729
	During	0.390	0.051	
F4: Right frontal	Before	0.379	0.044	0.590
	During	0.404	0.050	
T3: Left temporal	Before	0.298	0.045	0.329
	During	0.327	0.054	
T4: Right temporal	Before	0.332	0.036	0.692
	During	0.349	0.051	
P3: Left parietal	Before	0.403	0.048	0.689
	During	0.422	0.057	
P4: Right parietal	Before	0.397	0.041	0.414
	During	0.437	0.056	
O1: Left occipital	Before	0.373	0.039	0.304
	During	0.429	0.063	
O2: Right occipital	Before	0.432	0.073	0.757
	During	0.451	0.074	

**Table 5:** Effect of inhalation of volatile organic chemicals (VOCs) from *Chrysanthemum indicum* Linné on relative beta wave.

Site	Relative Beta Activity (μV^2^)			
	Inhalation	Mean	Standard Error	*p*-Value
Fp1: Left prefrontal	Before	0.198	0.035	0.619
	During	0.186	0.030	
Fp2: Right prefrontal	Before	0.186	0.032	0.683
	During	0.203	0.025	
F3: Left frontal	Before	0.221	0.024	0.349
	During	0.195	0.025	
F4: Right frontal	Before	0.212	0.021	0.435
	During	0.192	0.027	
T3: Left temporal	Before	0.240	0.032	0.518
	During	0.221	0.025	
T4: Right temporal	Before	0.240	0.026	0.306
	During	0.213	0.028	
P3: Left parietal	Before	0.221	0.023	0.462
	During	0.201	0.028	
P4: Right parietal	Before	0.226	0.022	0.192
	During	0.193	0.029	
O1: Left occipital	Before	0.247	0.024	0.225
	During	0.216	0.032	
O2: Right occipital	Before	0.219	0.033	0.194
	During	0.185	0.035	

**Table 6:** Effect of inhalation of volatile organic chemicals (VOCs) from *Chrysanthemum indicum* Linné on relative gamma wave.

Site	Relative Gamma Activity (μV^2^)			
	Inhalation	Mean	Standard Error	*p*-Value
Fp1: Left prefrontal	Before	0.108	0.024	0.851
	During	0.104	0.016	
Fp2: Right prefrontal	Before	0.112	0.022	0.883
	During	0.115	0.012	
F3: Left frontal	Before	0.110	0.021	0.343
	During	0.091	0.011	
F4: Right frontal	Before	0.097	0.015	0.590
	During	0.087	0.014	
T3: Left temporal	Before	0.128	0.025	0.548
	During	0.113	0.014	
T4: Right temporal	Before	0.123	0.024	0.358
	During	0.101	0.013	
P3: Left parietal	Before	0.104	0.018	0.452
	During	0.089	0.014	
P4: Right parietal	Before	0.103	0.017	0.134
	During	0.072	0.010	
O1: Left occipital	Before	0.127	0.022	0.301
	During	0.107	0.016	
O2: Right occipital	Before	0.105	0.024	0.537
	During	0.092	0.022	
